# The Landmark Series: Intraductal Papillary Mucinous Neoplasms of the Pancreas—From Prevalence to Early Cancer Detection

**DOI:** 10.1245/s10434-022-12870-w

**Published:** 2023-01-04

**Authors:** Tommaso Pollini, Paul Wong, Ajay V. Maker

**Affiliations:** Division of Surgical Oncology, Department of Surgery, University of California San Francisco, San Francisco, CA USA

## Abstract

Modern series report a prevalence of pancreatic cysts in the general population of up to 50% in prospective studies. Of these, about half will be pancreatic cystic neoplasms (PCNs) that have varying degrees of malignant potential. Intraductal papillary mucinous neoplasms (IPMNs) of the pancreas are the most common PCNs and are known predecessors of pancreatic adenocarcinoma. Critically, they are one of the only radiographically identifiable precursors of pancreatic cancer and thus provide an opportunity for early cancer detection and surgical resection with curative intent. The combination of high prevalence and potential for malignant degeneration underscore the relevance of discussing the best management of IPMNs and improving the existing standard of care. Landmark data on IPMN prevalence, guidelines, surveillance, biomarkers, and immune landscape are highlighted.

This article is structured as a toolkit of relevant landmark articles essential to current intraductal papillary mucinous neoplasm (IPMN) care. Four elements will be highlighted. First, the studies quantifying the prevalence of pancreatic cystic neoplasms (PCNs) and IPMNs in the general population are addressed. No longer considered a rare disease, providing an accurate estimate of the numbers of patients with IPMN frames the relevance of the topic. Since the definition and classification of IPMN was not formally adopted by the World Health Organization until 1996, modern-era studies have been selected. Second, the main clinical management guidelines are discussed in context. A concise description of the methodology used to develop the recommendations in each guideline is given, followed by direct comparison of management strategies in the different guidelines. Third, the evidence on surveillance discontinuation is discussed. The reason for highlighting this ongoing and timely debate is that non-resected IPMNs are currently considered for lifelong surveillance. Given the high prevalence of IPMNs and length of surveillance, the burden on patients and the health care system worldwide demands a reassessment of surveillance discontinuation for specific subsets of IPMNs. Fourth, due to the limitations and low specificity of current guidelines to identify appropriate patients for pancreatic resection more accurately, the main studies on new cyst biomarker panels to predict IPMNs at high-risk for cancer are discussed. The goal of molecular diagnostics of cyst fluid aims to reduce the number of unnecessary resections that are performed every year, while also providing information on the malignant potential of lesions under surveillance. A final comment on the role of the immune system in IPMN progression is given, as opportunities for early immunotherapeutic intervention could change the course of this disease.

## Prevalence in the General Population

### Study 1: Incidental Pancreatic Cystic Neoplasms in an Asymptomatic Healthy Population of 21,745 Individuals^1^

#### Methods

Chang et al. report on 25,300 healthy individuals undergoing abdominal computed tomography as part of a preventive screening campaign between 2003 and 2013 at Seoul National University Hospital Healthcare System Gangnam Center. To ensure that pancreatic cysts identified in the study were truly incidental, any patient with a history of pancreatic or gastrointestinal surgery were excluded, as were any patients with known or suspected pancreatic disease or abdominal symptoms. After imaging review by two specialized radiologists, any pancreatic cystic lesion over 5 mm in diameter was classified as either an IPMN, mucinous cystic neoplasm (MCN), or serous cystic neoplasm as appropriate.

#### Results

Of 21,745 patients included in the study, 457 (2.1%) incidental pancreatic cysts were identified, with a median size of 10 mm. The prevalence of pancreatic cystic lesions, and of IPMNs specifically, increased significantly with age, reaching 13.5% and 12.2%, respectively, in patients over 80 years of age.

### Study 2: Prospective Study on the Incidence, Prevalence, and 5-Year Pancreatic-Related Mortality of Pancreatic Cysts in a Population-Based Study^2^

#### Methods

Kromrey et al. included subjects who were enrolled in the Study of Health in Pomerania (SHIP). This was a prospective population-based cohort study in Northeastern Germany to rate the prevalence of diseases and to study correlations between risk factors. Of 1077 people with baseline abdominal magnetic resonance cholangiopancreatography, 686 accepted a 5-year follow-up and re-evaluation. Any pancreatic cystic lesion above 2 mm in diameter was noted during review by a single radiologist.

#### Results

The prevalence of pancreatic cysts at baseline was 49.1%. The majority of patients (63.6%) had cysts below 5 mm in diameter, and only 5.8% had a cyst over 10 mm in diameter. Prevalence and median cyst size increase with age, with people over 80 years of age having a 75.7% cyst prevalence and a median size of 6.8 mm. Of patients who did not demonstrate a pancreatic cyst at baseline and who were included in the 5-year follow-up, 48 showed a new cyst, with a weighted 5-year incidence of 12.9% (2.6% per year). While considering patients who had a cyst at baseline, 49.8% showed an increase in size after 5 years.

### Study 3: Prevalence, Incidence, and Risk of Progression of Asymptomatic Pancreatic Cysts in Large-Sample, Real-World Data^3^

#### Methods

Over 200 million patients were assessed using the IBM MarketScan administrative claims database, which is based on billed diagnosis and procedures from a variety of health plans in the US. Of these, 700,000 individuals without conditions that predispose patients to pancreatic cysts were included in the prevalence analysis.

#### Results

The prevalence of pancreatic cysts increased significantly with age and was 1.84% in patients 45 years or older, 3.0% in patients between 75 and 84 years of age, and 2.4% in patients 85 years of age or older. Standardized incidence grew over time from 6.3 to 11.4 per 10,000 people between 2010 and 2017, while the imaging rate only changed from 8.0 to 9.4%. The annual progression rate was 0.47%.

### Comment

The prevalence of PCNs in the general population ranges between 3 and 75%, the majority of which will be IPMNs. These three studies were included due to their large denominators and population or claims-based approach. The drastic difference in prevalence among these large studies is likely a consequence of the difference in methodology and imaging modalities. Depending on the inclusion criteria and how imaging revision is performed, the rate of identified cysts changes significantly. Chang et al. and Kromrey et al. each reviewed computed tomography (CT) and magnetic resonance imaging (MRI), respectively, looking for undiagnosed cystic lesions,^1,2^ whereas Schweber et al. relied on existing diagnoses.^3^ Autopsy studies have estimated the prevalence at about 24%. What emerges from these population studies is that PCN prevalence increases with age, rising to over 75% in patients 80 years of age or older. Notably, not all lesions will qualify for a diagnosis of IPMN, as even lesions below 5 mm in diameter are included in some of these studies, and perhaps half of these lesions will be IPMNs. These results are meaningful because every cyst will require assessment by a specialist, and likely be re-evaluated with expensive cross-sectional imaging to determine which cysts require surveillance, further examination, or surgery. Given the high prevalence of incidental IPMNs, screening of the general population for IPMNs is impractical and will result in millions of newly diagnosed cysts and significant patient anxiety. The vast majority of these cysts will be subcentimeter and with exceedingly low likelihood of malignancy. However, since the prevalence is so high, improved diagnostic tests are clearly necessary to establish low-risk cysts from high-risk cysts.

## Guidelines for Intraductal Papillary Mucinous Neoplasm (IPMN) Management

### 2015 American Gastroenterological Association Guideline on the Diagnosis and Management of Asymptomatic Neoplastic Pancreatic Cysts^4^

These guidelines were established by the American Gastroenterological Association (AGA) for the diagnosis and management of asymptomatic PCNs (Table 1).^4^ The AGA guidelines only address asymptomatic cysts and do not evaluate the impact of symptoms on the management of these cysts. The AGA guidelines were established following a systematic evaluation of the relevant evidence on the management of these pancreatic cysts. An expert consensus was acquired on clinically relevant questions regarding diagnosis and management. The conclusions of the systematic reviews are reported in the AGA’s technical review^5^ and were used by the AGA’s Clinical Practice Guideline Committee as the basis for the formal guidelines. Specifically, the recommendations use the Grading of Recommendations Assessment, Development and Evaluation (GRADE) framework.^6^ Notably, the committee graded all the evidence related to the management of pancreatic cysts to be very low in quality because most of the data were collected from case series. Thus, all their statements were considered conditional and dependent on each patient’s situation.

**Table 1:** Comparison of selected guidelines for intraductal papillary mucinous neoplasm (IPMN) management

	2015 American Gastroenterological Association (AGA) Guidelines	2017 International Association of Pancreatology (IAP) Revised Fukuoka Guidelines	2018 European Study Group on Cystic Tumors of the Pancreas Guidelines
Preoperative cyst diagnosis	NA	MRI is preferred over CT; EUS/FNA (cytology/cyst fluid analysis) performed for better diagnosis	MRI is preferred over CT; EUS/FNA (cytology/cyst fluid analysis) performed for better diagnosis
Biomarkers	NA	CA19-9, CEA, amylase, molecular biomarkers (i.e., KRAS, GNAS)	CA19-9, CEA, amylase, molecular biomarkers (i.e., KRAS, GNAS)
Non-operative surveillance	MRI/CT at 1 year, then every 2 years for 5 years total	< 1 cm: CT/MRI in 6 months, then every 2 years if no changes 1–2 cm: CT/MRI in 6 months for 1 year, then in 1 year for 2 years, then every 2 years 2–3 cm: EUS in 3–6 months then alternating EUS and MRI annually > 3 cm: MRI alternating with EUS every 3–6 months	EUS and/or MRI every 6 months for 1 year, then annually as long as surgically fit
Indications for surgery	Dilated main pancreatic duct, solid cystic component, cytology with high-grade dysplasia or invasive carcinoma	*Surgery*	
		High-risk stigmata: Jaundice (mass-related), enhancing mural nodule ≥ 5 mm, main pancreatic duct ≥ 10 mm	Absolute indications: Cytology with high-grade dysplasia or invasive carcinoma, main pancreatic duct dilatation > 10 mm, mural nodule > 5 mm, solid mass, jaundice (mass-related)
		*Proceed with EUS*	*Consider surgery*
		Worrisome feature: Pancreatitis, cyst ≥ 3 cm, enhancing mural nodule < 5 mm, thickened/enhancing cyst walls, main duct size 5–9 mm, abrupt change in caliber of pancreatic duct with distal pancreatic atrophy, lymphadenopathy, increased serum level of CA19-9, cyst growth rate ≥ 5 mm/2 years	Relative indications: Growth rate > 5 mm/year, CA19-9 > 37 U/mL, main pancreatic duct dilatation 5–9 mm, cyst diameter > 4 cm, symptoms of new-onset diabetes and acute pancreatitis, mural nodule < 5 mm
Frozen section	NA	Recommended: If margin-positive for invasive cancer or high-grade dysplasia, additional resection is warranted to obtain a negative margin. If low-grade dysplasia is present at margin, further resection is not necessary	Recommended: If margin-positive for invasive cancer or high-grade dysplasia, additional resection is warranted to obtain a negative margin. If low-grade dysplasia is present at margin, further resection is not necessary
Postoperative follow-up	Invasive cancer or high-grade dysplasia: MRI every 2 years No high-grade dysplasia or invasive cancer: no additional surveillance required	No increased risk for malignancy: CT/MRI every 6–12 months Higher risk for malignant progression: CT/MRI at least 2 times/year Invasive IPMN: Same follow-up strategy as PDAC	Low-grade dysplasia: EUS and/or MRI every 6 months for 1 year, then annually as long as surgically fit High-grade dysplasia or MD-IPMN: EUS and/or MRI every 6 months for the first 2 years, then annual surveillance Invasive IPMN: same follow-up strategy as PDAC

*NA* not available, *MRI* magnetic resonance imaging, *CT* computed tomography, *EUS* endoscopic ultrasound, *FNA* fine-needle aspiration, *CEA* carcinoembryonic antigen, *PDAC* pancreatic ductal adenocarcinoma, *MD-IPMN* main duct intraductal papillary mucinous neoplasm, *IPMN* intraductal papillary mucinous neoplasm

### 2017 International Association of Pancreatology (IAP): Revisions of International Consensus Fukuoka Guidelines for the Management of IPMNs of the Pancreas^7^

The original ‘Sendai’ guidelines on the management of IPMNs and MCNs were created by the International Association of Pancreatology (IAP) at the 11th Congress of the IAP in Sendai, Japan, and published in 2006.^8^ These were consensus guidelines related to the classification, preoperative evaluation, surgical indications, resection technique, histology, and surveillance of IPMNs and MCNs. Subsequently, these recommendations were updated in 2012 in Fukuoka, Japan, and again in 2017. The latest version of the IAP guidelines distinguished operative criteria for branch duct IPMNs (BD-IPMNs), as it built upon the classifications of ‘worrisome features’ and ‘high-risk features’. Mucinous cystic neoplasms (MCN) were excluded from this 2017 revision.

In this updated version of the Fukuoka guidelines, as they are called, the framework of recommendations follows that of prior iterations of the IAP guidelines. Namely, the expert consensus provides recommendations on the classification, preoperative evaluation, surgical indications, methods of resection and other treatments, histological aspects, and surveillance of IPMNs.

### 2018 European Study Group on Cystic Tumors of the Pancreas: European Evidence-Based Guidelines on Pancreatic Cystic Neoplasms^9^

These guidelines were established as a joint initiative of several European scientific societies for the management of PCNs. This rendition of the European guidelines replaced the prior European consensus statement guidelines on PCN published in 2013.

A methodology committee consisting of gastroenterologists, surgeons, radiologists, oncologists, endoscopists, and basic scientists was compiled. Subsequently, systematic reviews were performed by experts in their respective topic areas to encompass all the available evidence regarding predetermined clinical questions. This literature search only included randomized trials, observational cohort studies with more than 20 patients, and systematic reviews on PCN. Following this systematic review of the literature, recommendations were established by expert consensus and supplemented with ratings for the quality of evidence using the GRADE framework.^6^ Statements regarding the strength of the recommendation were added. To finalize the guidelines, these recommendations were reviewed at a plenary meeting of the European Study Group on Cystic Tumors of the Pancreas, and at least 75% consensus was required to establish the recommendation.

### Guideline Comparisons for Surveillance and Operative Intervention

#### Non-operative Surveillance

For non-operative surveillance, all three sets of guidelines state that MRI is the preferred modality over CT because MRI does not expose patients to radiation and is better able to confirm the structural relationship between the cyst and pancreatic duct.

At baseline, the revised Fukuoka guidelines suggest that BD-IPMN patients without high-risk stigmata or worrisome features (> 3 cm, enhancing mural nodularity or cyst wall, main duct dilation, stricture, lymphadenopathy, elevated CA19-9, significant growth rate, jaundice, or symptoms) undergo short interval (3–6 months) pancreatic MRI/MRCP or CT to confirm stability. However, unlike the European and AGA guidelines, the revised Fukuoka guidelines provide recommendations on subsequent surveillance according to cyst size, as summarized in Table 1.

For patients without an indication for immediate resection, the European guidelines recommend a 6-month follow-up the first year, with subsequent annual follow-ups as long as no risk factors are present that would prompt surgery. In addition, in patients with only relative indications for resection, older patients, and those with severe comorbidity, continual 6-month follow-up is suggested.

In cases where there are not indications for resection, the AGA guidelines recommend that patients with cysts < 3 cm without a solid component or dilated pancreatic duct receive MRI surveillance in 1 year, followed by every 2 years, for a total period of 5 years if there are no changes in size or characteristics. The recommendation of the AGA guidelines to stop surveillance after 5 years is different from the European and revised Fukuoka guidelines, which suggest indefinite surveillance of IPMNs as long as the patient is fit for surgery because the risk of IPMN progression increases over time.

#### Indications for Surgery

The indications for surgery in these guidelines are listed in detail in Table 1. For main duct IPMN (MD-IPMN), the recommendation from the IAP guidelines is surgical resection for all surgical candidates with main pancreatic duct diameter > 10 mm, jaundice, or mural nodules; however, the European guidelines recommend that all patients with MD-IPMN should undergo resection if they are fit for surgery. Both the European and IAP guidelines describe the value of frozen section biopsies to determine the extent of resection. In both guidelines, additional resection is warranted to obtain a negative margin if invasive cancer or high-grade dysplasia is found at the initial resection margin, whereas further resection is not necessary if low-grade dysplasia is discovered at the margin.

From the AGA guidelines, the general indications for surgery for asymptomatic pancreatic cysts include both a solid component and a dilated pancreatic duct and/or concerning features on endoscopic ultrasound (EUS) and fine-needle aspiration (FNA). The concerning features on EUS/FNA include solid component, dilated duct, or positive cytology. Because the presence of these concerning features increases the risk for malignancy, surgery is recommended for these patients as it would reduce the risk of mortality from carcinoma. In addition, AGA guidelines suggest that surgical candidates be referred to centers that have demonstrated expertise in pancreas surgery in order to decrease immediate postoperative mortality and optimize long-term survival.

For BD-IPMN, a generally conservative management strategy is suggested in the IAP guidelines for patients without features that predict invasive carcinoma or high-grade dysplasia. The IAP guidelines also describe the absolute indications for resection of BD-IPMNs, which are positive cytology for high-grade dysplasia and the presence of mural nodules ≥ 5 mm. Notably, cyst size alone is not considered an appropriate parameter to indicate surgery; however, cysts > 2 cm in patients < 65 years of age are considered as candidates for resection given the lifelong cumulative risk of high-grade dysplasia and invasive carcinoma. The European guidelines share the same absolute indications as the IAP guidelines in addition to the presence of jaundice and solid mass. Relative indications for surgery listed in the European guidelines prompt consideration of surgery, while the equivalent worrisome features in the IAP guidelines require EUS to be performed, and only to consider surgery if specific features are present (Table 1).

### Comment

There are now over 10 guidelines by different societies, specialty groups, and geographies. The three guidelines included in this article are the most widely used among pancreatologists worldwide. AGA guidelines have not been updated since 2015 and have found limited adoption by the surgical oncology community, particularly in regard to repeat use of EUS and surveillance cessation. The Sendai/Fukuoka/International guidelines have been updated three times, now almost exclusively focused on IPMN. As outlined, they rely for the most part on expert opinion and are therefore at the bottom of the classical pyramid for evidence-based medicine.^10^ While most clinicians will endeavor to follow at least one of these guidelines, there remains significant differences between guideline recommendations and clinical practice.^11^ An added layer of complexity when navigating the guidelines in clinical practice is differences in guideline recommendations and terminology, and these issues hamper their use in the real world. Future updates to these guidelines should endeavor to provide a single universally accepted version, distinguishing MCN from IPMN, and incorporating methodologies, i.e. radiomics and molecular diagnostics, that improve their positive predictive value. The current guidelines are very sensitive and few invasive cancers are being surveilled, however they have low specificity. It is estimated that using these guidelines, approximately 75% of IPMN resections will be for low-risk lesions that could otherwise have been surveilled.^12^

## Long-Term Surveillance of Presumed Branch Duct (BD)-IPMN

The vast majority of IPMNs are currently kept under surveillance. Considering the increasing prevalence of these lesions and the median age at diagnosis of 65 years, the burden of lifelong surveillance on patients and health care systems has risen drastically in recent years. In 2015, the AGA guidelines recommended surveillance discontinuation after 5 years if no changes are observed. This recommendation prompted the publication of a large number of retrospective observational series,^13^ aimed at assessing the risk of malignant degeneration after 5 years of stability for presumed BD-IPMN. In their study, Lawrence et al. reported a higher incidence of pancreatic cancer (31 per 100,000 per year) in those BD-IPMNs that were stable for the first 5 years of surveillance compared with the expected age-adjusted incidence (7 per 100,000 per year).^14^ Similarly, Oyama et al. reported a 3% and 12% incidence of pancreatic cancer in presumed BD-IPMNs at 10- and 15-year follow-up, respectively.^15^ In a similar analysis of over 1700 presumed BD-IPMNs, Han et al. found a 19% and 35% cumulative risk of developing a worrisome feature or high-risk stigmata at 5- and 10-year follow-up, respectively.^16^ These results highlight the fact that having a BD-IPMN in general confers a higher risk of developing a pancreatic cancer even after long-term surveillance. It may be that surveillance discontinuation should be tailored to the age of the patient. Marchegiani et al. reported a standardized incidence ratio (SIR) of pancreatic cancer of 3.8 (95% confidence interval [CI] 0.77–11.20) in patients with a presumed BD-IPMN without worrisome features or high-risk stigmata at diagnosis and after 5 years of surveillance over 65 years of age.^17^ This SIR was not significantly different from that of the age-matched general population.^18^ Therefore, the added risk associated with the presence of a stable BD-IPMN in certain age groups might be non-significant.

### Comment

As reported in these studies, there is a non-zero risk of developing suspect features or a pancreatic cancer even after 5 years of surveillance. For that matter, there is a risk of developing pancreatic cancer in a remnant gland even after resection of an IPMN. Thus, one can consider IPMN as a field defect in the gland. Therefore, it is not possible to currently support the recommendation to discontinue surveillance in presumed BD-IPMNs that are stable for 5 years. Further research will be needed to provide suitable targets for surveillance discontinuation, although the data do support extending the time interval between scans once stability has been determined, and potentially focusing surveillance on certain age groups at diagnosis.

## Biomarkers for the Diagnosis and Risk Stratification of IPMNs

### Study 1: A Multimodality Test to Guide the Management of Patients with a Pancreatic Cyst^19^

#### Methods

Springer et al. analyzed the cyst fluid of 862 patients collected during surgery (85%) or EUS (15%). Purified DNA was analyzed for mutations in the following genes, *KRAS, GNAS, RNF43, CDKN2A, CTNNB1, SMAD4, TP53, VHL, BRAF, NRAS*, and *PIK3CA* using multiplex polymerase chain reaction (PCR) and MiSeq or HiSeq sequencing. Loss of heterozygosity (LOH) was determined in specific tumor suppressor genes (*CDKN2A, RNF43, SMAD4, TP53*, or *VHL*) and aneuploidy evaluated with FastSeqS. Carcinoembryonic antigen (CEA) and vascular endothelial growth factor (VEGF)-A concentration in the cyst fluid was also assessed using a Bio-Plex 200 bead-based immunoassay. Samples were labeled using the diagnosis from final pathological examination. A machine learning algorithm was used to build a composite marker termed CompCyst.

#### Results

The resulting test identified mucinous cysts with 77% sensitivity and 86% specificity, compared with 80% sensitivity and 55% specificity from the preoperative clinical diagnosis. Serous cystic neoplasms were classified with 65% sensitivity and 99% specificity, improving from 18% sensitivity and 99% specificity with preoperative clinical diagnosis. Interestingly, both adenocarcinoma with cystic degeneration and pancreatic neuroendocrine tumors were classified with a higher sensitivity (71% vs. 58% and 86% vs. 71%, respectively) but lower specificity (90% vs. 96% and 92% vs. 99%, respectively) compared with the preoperative clinical diagnosis. After labeling patients as cleared from work-up and surveillance, requiring surveillance, or recommending surgery, CompCyst improved the accuracy in identifying patients requiring discharge from 19 to 60%, and of those requiring surveillance from 34 to 48%. CompCyst also improved the identification of patients requiring surgery by 2% (from 89 to 91%), compared with standard-of-care diagnosis.

### Study 2: Cyst Fluid Biosignature to Predict Intraductal Papillary Mucinous Neoplasms of the Pancreas with High Malignant Potential^20^

#### Methods

After previous work on the identification of ideal biomarkers for the identification of high-risk IPMNs,^21–23^ Maker et al. analyzed cyst fluid from an international cohort of patients. In their study, they used a combination of quantitative PCR (qPCR) [*IL1β, MUC-1, MUC-2, MUC-4, MUC-5ac, MUC-7, PTGER2, PTGS-1, PGE2-R, KRAS, GNAS, GAPDH, RPLP0, TP63, ERBB2, PTGES2*] and Sanger sequencing (*GNAS* codon 201 and *KRAS* codon 12 and 13). The resulting expression values and mutational analysis were combined using a support-vector machine and a Lasso-penalized logistic regression to identify the best combination maximizing the area under the curve (AUC) to predict the diagnosis of high-grade dysplasia or invasive cancer versus low-risk IPMNs

#### Results

The best combination of biomarkers to predict high-risk IPMNs was composed of *IL1β*, *MUC4,* and *PTGES2*, as calculated using the following formula (Eq. 1):

1 $$y=0.37+\left(-0.06*IL1\beta \right)+\left(-0.01*MUC4\right)+(-0.50*PTGES2)$$

The above biosignature had an accuracy of 86%, which was an improvement from the accuracy of available guidelines, being 50%, 76%, and 60% for the IAP, AGA, and American College of Radiology (ACR) guidelines, respectively.^24^

### Study 3: Incremental Value of DNA Analysis in Pancreatic Cysts Stratified by Clinical Risk Factors^25^

#### Methods

After assessing the value of DNA analysis in pancreatic cysts,^26,27^ Farrell et al. analyzed cyst fluid from 478 patients collected during EUS, of whom 209 had a final pathological diagnosis (either by surgical resection or biopsy/cytology). A commercially available test (PancraGEN, Interpace Diagnostics, Pittsburgh, PA, USA) that combines oncogene mutation analysis (*KRAS* codon 12 and 13) and LOH for tumor suppressor genes (*VHL, OGG1, PTEN, MX11, TP53, CDKN2A, RNF43, NME1, SMAD4, DCC, PSEN2, TFF1, CMM1, LMYC, MCC, APC*) was used. Presence of worrisome features or high-risk stigmata, as defined by the 2012 IAP Guidelines,^28^ were included in the analysis. One or more DNA abnormalities (defined as elevated DNA quantity, *KRAS* mutation, or LOH) were assessed in combination with the presence of one or more worrisome features, high-risk stigmata, or both, with regard to malignancy-free survival.

#### Results

An elevated DNA quantity, KRAS mutation, and LOH had a 73%, 88%, and 87% specificity for malignancy, respectively. The presence of two DNA abnormalities (among elevated DNA quantity, KRAS mutation, or LOH) had a 95–99% specificity, with 60 patients (12.5% of the entire study population) in this category. In patients with one or more high-risk stigmata and in those without any worrisome features or high-risk stigmata, the presence or absence of any DNA abnormalities did not alter malignancy-free survival. In patients with one or more worrisome features but without high-risk stigmata, the presence of two or more DNA abnormalities decreased malignancy-free survival (hazard ratio [HR] 4.9, *p* < 0.002), while the presence of one DNA abnormality did not alter malignancy-free survival.

### Study 4: Preoperative Next-Generation Sequencing of Pancreatic Cyst Fluid is Highly Accurate in Cyst Classification and Detection of Advanced Neoplasia^29^

#### Methods

Building on previous publications,^30,31^ Singhi et al. analyzed cyst fluid samples obtained from EUS/FNA of 595 patients. Diagnosis was available for 102 patients (17%) and was based on pathological examination of the surgical or cytopathology specimen. A next-generation sequencing (NGS) assay was developed assessing genes that are known to be frequently mutated or deleted in pancreatic cysts (*KRAS, GNAS, NRAS, HRAS, BRAF, CTNNB1, TP53, PIK3CA, PTEN* and *AKT1*) in combination with Sanger sequencing of *VHL*.

#### Results

Based on 102 patients with available pathological diagnosis (56 IPMNs, 10 MCNs, 3 serous cystadenomas [SCAs], 9 cystic pancreatic neuroendocrine tumors [cPanNETs], and 24 non-neoplastic cysts), NGS detection of KRAS and/or GNAS mutations had an 89% sensitivity and 100% specificity for IPMNs and MCNs. The combination of mutations in *KRAS* and/or *GNAS*, and alterations of *PTEN*, *TP53* and/or *PIK3CA*, had a 79% sensitivity and 96% specificity for HGD/invasive cancer in mucinous lesions. The addition of mutant allele frequency, measured as the number of reads of the mutant allele versus the wild-type allele and reported as a percentage, increased sensitivity and specificity to 89% and 100%, respectively.

### Comment

Several biomarkers have been identified to aid in IPMN diagnosis and malignant potential based on differential expression of oncogenes, tumor suppressor genes, glycoproteins, immune modulators, proteins, and DNA/RNA/miRNA.^32^ The studies included in this article represent a summary of gene expression and NGS approaches using IPMN cyst fluid. It is clear that KRAS/GNAS mutations are associated with mucinous cystic neoplasms, including IPMNs, and that there are individual genes that are associated with high-grade dysplasia and invasive cancer when either mutated or differentially expressed. Other authors have explored the use of proteomic signatures with the landmark representative study, including a panel of MMP9, CA72.4, sFASL, and IL-4.^33^ Additionally, other groups have evaluated different biofluids, including saliva, serum, and urine;^34,35^ however, we believe that cyst fluid enables evaluation of shed IPMN cells/DNA at their source. Building on their prior work that identified high-risk mutations and LOH, Springer et al. performed an extensive assessment of the genetic phenotype of cystic lesions using multiple platforms. After analyzing these results with a machine learning-based approach, the accuracy increase over traditional radiological assessment was 2% for patients requiring surgery, and the score was able to improve prediction of unnecessary resection. Farrell et al. were able to better define the population for which the assay they studied was most useful (IPMNs with worrisome features), and a recent update to the study by Singhi et al. has validated the genes in their panel as being associated with cyst diagnosis and high-risk disease, and extended the analysis to EUS-obtained specimens.^29^ Maker et al. harnessed differential gene expression levels to create a biosignature utilizing a single and low-cost platform to predict high-risk IPMNs, with a high level of accuracy, and current work has built out the platform using a microfluidic plate. The field waits for these molecular diagnostics to be extensively deployed in clinical practice. Diagnostic predecessors to these most recent landmark papers have been available, for example the panel utilized by Farrell et al., but due to limitations of low sensitivity as shown in the PANDA trial, discordance with surgical pathology, and concern for practical utility^36^ have not been included in standard-of-care work-up or guidelines. We think the papers reviewed in this section demonstrate that the field has significantly advanced during this time, that our understanding of the molecular basis of IPMN dysplasia has advanced, and that molecular diagnostics will become indispensable in the work-up of our patients with IPMNs in the not so distant future.

A final note about the tumor immune microenvironment of IPMNs. There is no doubt that progression of IPMN dysplasia is associated with proinflammatory cytokines and a T helper (Th)1/Th2 immune response.^21,23^ However, as lesions progress from low-grade dysplasia to invasive cancer, they seem to lose their cytotoxic T-cell infiltrates in favor of a suppressive immune microenvironment.^37^ We believe this change in the milieu is reflected in the molecular changes of the summarized panels, and further work may lead us to manipulate the tumor immune microenvironment to halt or potentially reverse IPMN progression.

## Conclusions

Due to the widespread availability and increased use of high-resolution cross-sectional imaging, incidental PCNs are increasing in incidence. Certainly, the numbers of patients that are referred to pancreatic surgery clinics have steadily increased over the years as a result. The prevalence of PCNs is more common in the general population than previously considered and increases with age, rising to over 75% in patients over 80 years of age in selected populations. About half of PCNs will be IPMNs, thus radiographic screening is impractical, although once identified, surveillance is necessary. When to stop this surveillance is difficult to determine and may be abbreviated in older individuals, but certainly will need to be more than 5 years in the absence of improved prognostic tools for the general population. Radiographic characteristics remain the main components of current treatment guidelines and there are significant differences between guideline recommendations and clinical practice. Future updates to these guidelines will benefit from an improved body of evidence and should endeavor to provide a single universally accepted version, distinguish MCNs from IPMNs, and incorporate methodologies that improve their positive predictive value. Cyst fluid molecular diagnostics have shown promise in differentiating low-grade dysplastic IPMNs from high-grade and invasive IPMNs, and there is now evidence that dysplasia is associated with distinct changes in the tumor immune microenvironment.
